# Implementation of an Intersectoral Program to Eliminate Human and Canine Rabies: The Bohol Rabies Prevention and Elimination Project

**DOI:** 10.1371/journal.pntd.0001891

**Published:** 2012-12-06

**Authors:** Stella Marie D. Lapiz, Mary Elizabeth G. Miranda, Romulo G. Garcia, Leonida I. Daguro, Meydalyn D. Paman, Frederick P. Madrinan, Polizena A. Rances, Deborah J. Briggs

**Affiliations:** 1 Office of the Provincial Veterinarian, Capitol Annex, Tagbilaran City, Philippines; 2 Global Alliance for Rabies Control, Manhattan, Kansas, United States of America; 3 Provincial Health Office, Capitol Annex, Tagbilaran City, Philippines; Swiss Tropical and Public Health Institute, Switzerland

## Abstract

**Background:**

The province of Bohol, located in the Visayas islands region in the Philippines has a human population of 1.13 million and was the 4th highest region for human rabies deaths in the country, averaging 10 per year, prior to the initiation of the Bohol Rabies Prevention and Elimination Project (BRPEP).

**Aims:**

The BRPEP was initiated in 2007 with the goal of building a sustainable program that would prevent human rabies by eliminating rabies at its source, in dogs, by 2010. This goal was in line with the Philippine National Rabies Program whose objective is to eliminate rabies by 2020.

**Methods:**

The intersectoral BRPEP was launched in 2007 and integrated the expertise and resources from the sectors of agriculture, public health and safety, education, environment, legal affairs, interior and local government. The program included: increasing local community involvement; implementing dog population control; conducting mass dog vaccination; improving dog bite management; instituting veterinary quarantine; and improving diagnostic capability, surveillance and monitoring. Funding was secured from the national government, provincial, municipal and village units, dog owners, NGOs, the regional office of the WHO, the UBS Optimus Foundation, and the Global Alliance for Rabies Control. The BRPEP was managed by the Bohol Rabies Prevention and Eradication Council (BRPEC) under the jurisdiction of the Governor of Bohol. Parallel organizations were created at the municipal level and village level. Community volunteers facilitated the institution of the program. Dog population surveys were conducted to plan for sufficient resources to vaccinate the required 70% of the dogs living in the province. Two island-wide mass vaccination campaigns were conducted followed by “catch up” vaccination campaigns. Registration of dogs was implemented including a small fee that was rolled back into the program to maintain sustainability. Children were educated by introducing rabies prevention modules into all elementary schools in Bohol. Existing public health legislation at the national, provincial, and municipal level strengthened the enforcement of activities. A Knowledge, Attitude and Practices (KAP) survey was conducted in 2009 to evaluate the educational knowledge of the population. Increased surveillance was instituted to ensure that dogs traveling into and out of the province were vaccinated against rabies. Human and animal cases of rabies were reported to provincial and national authorities.

**Key Results:**

Within the first 18 months of the BRPEP, human rabies deaths had decreased annually from 0.77 to 0.37 to zero per 100,000 population from 2007–2009. Between October 2008 and November 2010 no human and animal cases were detected. Increased surveillance on the island detected one suspected human rabies case in November 2010 and one confirmed case of canine rabies in April 2011. Two mass vaccination campaigns conducted in 2007 and 2008 successfully registered and vaccinated 44% and 70% of the dogs on the island. The additional surveillance activities enabled a mobilization of mop up vaccination activities in the region where the human and canine case was located. Due to the increased effective and continuous surveillance activities, rabies was stopped before it could spread to other areas on the island. The program costs totaled USD 450,000. Registration fees collected to maintain the program amounted to USD 105,740 and were re-allocated back into the community to sustain the program.

## Author Summary

The Province of Bohol, Philippines has eliminated dog and human rabies in less than three years by empowering the community and implementing an intersectoral strategy. In 2006, Bohol ranked 4th highest in the Philippines for human rabies, averaging 10 deaths per year. Launched in 2007, the program utilized a social awareness campaign, dog population control, mass dog vaccination campaigns, improved dog bite management and veterinary quarantine, a new diagnostic laboratory, expanded surveillance, and the inclusion of education modules into the school curriculum. Improving community compliance to existing national and provincial rabies laws and engaging volunteers to help conduct the project was a key to success. The program, led by the Governor of Bohol, was administered through a group of departments working together at a provincial and local level, and supervised through the Office of the Provincial Veterinarian. Financial support came through the Governor and several NGOs including the Global Alliance for Rabies Control. The program is self-sustaining, through a small dog registration fee fed back into the program, through the continuing education of children in their classrooms, and through the dedicated efforts of over 15,000 staff and volunteers throughout the island.

## Introduction

More than 99% of all global human rabies deaths occur as a result of being exposed to infected dogs. In the Philippines, as in most Asian countries, canine rabies is endemic and poses a particularly perilous risk for persons living in rural areas [1]. The number of human deaths attributed to rabies has not changed significantly in the past decade in the Philippines with an average of 250 reported annually between 1999–2009 [2]. As with other Asian countries, almost every human death in the Philippines occurs because the patient failed to seek post-exposure prophylaxis (PEP) after exposure due to a lack of educational awareness or, less often because the PEP received did not follow the WHO recommended protocol [3]. In 1991, Fishbein et al reported that the elimination of canine rabies in the Philippines would prevent human rabies and result in an estimated cost savings of 25 million US dollars annually with the program costs being recuperated within 4 to 11 years [4]. This cost-benefit study provided evidence that the most effective strategy to prevent human rabies in a canine rabies endemic country would be to eliminate rabies at the source of infection, i.e. the dog population.

Program sustainability is a critically important issue for all public health programs, but especially for resource-poor countries with limited budgets and many problems to resolve. Thus, a successful rabies prevention and control program must be built around the strengthening of intersectoral collaboration and cooperation between several public health components [5]. In 2007, the provincial government in Bohol made a commitment to eliminate canine rabies throughout the province by developing a strategic plan that would involve all of the stakeholders required and would ultimately be sustainable once rabies was eliminated.

The province of Bohol is located in central Visayas Region, Philippines and prior to 2007, was among the top ten provinces in the country for human rabies deaths, averaging 10 deaths (0.77 per 100,000 population) per year. Of the reported human rabies cases in 2007–2008, 25% occurred in children less than 15 years old after being bitten by an infected dog [2]. In order to control canine rabies and eventually eliminate human rabies deaths in Bohol province, strategic plans for the Bohol Rabies Prevention and Elimination Program (BRPEP) were developed by integrating expertise and resources from the sectors of agriculture, human health, education, legal and finance. The BRPEP was initiated in 2007 by the Provincial Government of Bohol and was spearheaded by the Office of the Provincial Veterinarian (OPV) and the Provincial Health Office (PHO). The ultimate goal of the BRPEP was to prevent human rabies by eliminating the disease at its source, in dogs, by 2010 and to ensure sustainability once rabies was eliminated, so that if rabies were re-introduced to the island, the ongoing surveillance would quickly detect the outbreak and ensure proper follow-up. The BRPEP was consistent with the Philippine National Rabies Program (PNRA), enacted by the House of Senate in May 2007 that set a goal of eliminating human and dog rabies throughout the country by 2020. The BRPEP utilized the PNRA's national policies and strategies for the control and elimination of human and animal rabies, to initiate and conduct the project.

From the outset of the project, the BRPEP was developed and executed as an intersectoral program including: Local community involvement; communication management; dog population control; mass dog vaccination; dog bite management; veterinary quarantine; and improved diagnostic capability, surveillance and monitoring. Funding for the BRPEP program was secured through many avenues including: Cost-sharing activities from the national government, provincial, municipal and village (or barangay) local government units; dog owners; local non-government organizations; the regional World Health Organization (WHO) office, the UBS Optimus Foundation, and the Global Alliance for Rabies Control, a not-for-profit organization focused on rabies control and prevention that worked closely with the OPV to bring technical support and coordination to the project. Although securing external funding was a key component of eliminating canine rabies, it was also critical to build the program in a sustainable manner to ensure that after canine rabies elimination, the program would not fail due to dwindling resources as occurred in Bohol during a previous effort to eliminate canine rabies. In 2000, seven years prior to the launch of the BRPEP, the first province-wide rabies mass dog vaccination program had been conducted on Bohol in conjunction with a regional initiative. This earlier program had been directly supported by the national government and had utilized only the existing veterinary service staff. The following year, in 2001, the dog vaccination coverage on Bohol had dropped to 2% and no allocation had been made for the purchase of vaccine the succeeding years.

This article focuses on how the BRPEP was developed and implemented over a three year period, from 2007–2010, in Bohol, Philippines and discusses specific intersectoral strategies applicable to establishing sustainable rabies control and prevention programs in resource-poor countries.

## Methods

### Project Area

**Figure 1:**
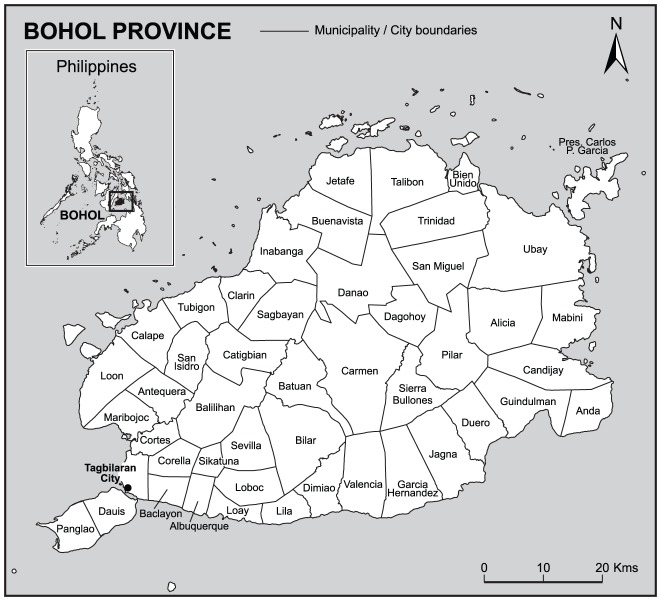
Map of the province of Bohol Philippines. BOHOL FACTFILE. Total Population: 1,139,130. Total Households: 152,324. Total Land Area: 11,726 hectares. 47 municipalities, 1 city & 1,109 barangays or villages. 81 offshore islands & islets. 30 coastal municipalities and 304 coastal barangays. Annual per capita income: 16,478 PHP (378 US$). Annual per capita expenditure: 14,364 PHP (378 US$). Annual average family income: 77,291 PHP (1,770 US$). Annual average family expenditure: 66,907 PHP (1,534 US$). PHP = Philippine Pesos.

The island province of Bohol is the 10th largest island in the Philippines (Figure 1). The total human population is 1,139,130 living in 152,324 households [6]. The economic drivers are eco-cultural tourism and agro-industrialization. The annual average family income is 77,291 Philippine Pesos (PHP) (1,770 US$) and the annual average family expenditure is 66,907 PHP (1,534 US$). Annual per capita income is 16,478 PHP (378 US$) and annual per capita expenditure is 14,364 PHP (329 US$).

### Program Management

At the provincial level, the BRPEP was managed by the Bohol Rabies Prevention and Eradication Council (BRPEC) under the jurisdiction of the Governor. Canine rabies prevention and elimination was coordinated by the Provincial Veterinarian while human rabies prevention and elimination was managed by the Provincial Health Officer. The BRPEC administered overall implementation, formulated proposals, measures and strategies that would ensure the implementation and sustainability of the BRPEP. This body also recommended the enactment of support legislation, policies and directives to strengthen the program and provided timely reports to program partners and the general public.

Parallel organizations to the BRPEP were created at the municipal and barangay (villages) local government units (LGU) namely the Municipal Rabies Prevention and Elimination Council (MRPEC) and the Bantay Rabies sa Barangay (BRB) or the “Rabies Watchers”. The MRPEC assumed the same roles and functions at their areas of jurisdiction in accordance with the BRPEP. The BRB ensured implementation of the program at the community level, arranged mass vaccination campaigns in their areas and compiled a master list of dogs and dog owners.

### Legal Framework for the Program

The legal framework for implementing a rabies prevention and control program in the Philippines was already in place at the inauguration of the BRPEP and included several national regulations: Republic Act No. 8485 known as the Animal Welfare Act was enacted in 1998; Philippine Republic Act No. 9482 was enacted on 25 May 2007 and is identified as the Anti-Rabies Act of 2007 [7]. The Philippine Republic Act was approved by the national government to be implemented at the LGUs. At the local level, the provincial Governor promulgated the Provincial Ordinance No. 2007-012 “Strengthening the Bohol Rabies Prevention and Eradication Program” that was approved on 10 July 2007 by the Sangguniang Panlalawigan or the local legislative body. This ordinance stipulated the establishment of the implementing bodies (BRPEC, MRPEC and BRB) at the provincial, municipal and barangay level. Membership of the municipal councils was designated by the Mayors and brought together representatives of the national offices (Departments of Agriculture, Health, Education, Interior and Local Government, Philippine National Police, Agricultural Training Institute and the Philippine Information Agency), the provincial offices (provincial veterinarian, health , tourism, legal, social welfare and agriculture), as well as representatives of several NGOs, league of elected and appointed officials and the local media group. BRB membership was designated by the barangay captains.

The local legislation defined the roles and responsibilities of the councils including: The organization of dog and dog owner registration; collection of registration fees; elimination of stray dogs (defined in the national law as any dog leaving its owner's place or premise and that is no longer in the effective control of the owner); dog vaccination; surveillance of human and animal rabies and dog bite incidents; settlement of disputes/agreements between bite victims and dog owners; and promotion of responsible dog ownership. To ensure dissemination of information and understanding of the supporting legislation and to promote program advocacy throughout the entire province, orientation and paralegal training sessions were conducted for 7,763 BRB volunteers.

### Communication Management

All communications regarding the BRPEP were managed in a coordinated manner to ensure integration of all components of the program as it progressed through the development, initiation, implementation, maintenance and sustainability phases. It was deemed critically important from the beginning of the BRPEP to facilitate understanding, cooperation, and support among stakeholders including the citizens living on Bohol, medical professionals, volunteers and paid employees implementing the program as well as government officials and funding agencies. Information was therefore delivered to the beneficiaries at the grass roots level as well as to government agencies overseeing the project and funding agencies supporting the project. The Provincial as well as the Municipal/City Rabies Task Forces designated key spokespersons authorized to dispense information and answer questions relevant to their areas of jurisdiction.

As part of the communications strategy, a BRPEP handbook was developed, published and distributed to every municipal rabies council to serve as a reference for all field units working within the program [8]. The handbook was designed to provide a logical web of operational activities among inter-agency stakeholders and a common approach in controlling rabies within the province. Moreover this document provided clear and distinct roles and responsibilities at various management levels from the national level down to the barangays and individual households. It also included vital information regarding the disease and its epidemiology. It outlined the goals and objectives of the BRPEP, strategies of implementation, program management, and information on legislation and issuances as well as contact phone numbers in case of questions about the program. Additionally, the BRPEP handbook included an example of an annual operational plan including the report forms and the monitoring format. A Barangay Handbook with simplified standard operating procedures written in the local dialect was also distributed to each of the 1,109 BRBs.

### Information and Educational Campaign (IEC)

A two-pronged social mobilization plan, including a ‘Community-focused program’ and a ‘School-based education program’, was launched as a first step to increase awareness and enhance community participation and support. Components of the IEC included discussions on rabies as a disease, its epidemiology, and its prevention and control, the Bohol Program in general and related national and municipal rabies ordinances as they supported the program implementation and responsible pet ownership.

The community program concentrated on campaigns using tri-media (television, radio, newspapers), display of posters and banners in strategic areas, distribution of flyers and other materials, public hearings of local ordinances and hosting of municipal and barangay symposia, meetings and seminars. Educational campaigns were also conducted at various government offices and in churches. Philippine National Rabies Awareness Month in March, and World Rabies Day, held annually on September 28 were both observed to remind people of the continual threat of rabies and the importance of the program to eliminate rabies on Bohol.

The School-based educational program, designed to improve awareness about rabies prevention, was developed and implemented in close supervision with the Department of Education and in coordination with the Department of Health and other member agencies of the BRPEC. The integration of rabies education into the school curriculum was initially developed by the Department of Health's National Rabies Control Program in 2006. In 2008, this program was piloted in Bohol beginning with round table discussions with teachers, followed by intensive planning, a workshop to develop lesson plans, orientation/training of teachers, and testing of the developed lesson plans for 6 months in the municipality of Corella during the 2008–2009 school year. In 2009, they were integrated into the curriculum of all 962 public elementary schools in Bohol. Educational activities for the children included: Incorporation of rabies modules into various subjects in the public elementary school curriculum; creation of “Rabies Scouters” (boy and girl scouts who have successfully completed a rabies and responsible pet ownership training program); creation of a campaign slogan to encourage responsible pet ownership; conducting fun educational events to celebrate the bond between children and pets. Other forms of campaigns promoting responsible pet ownership were also adopted in the city/municipalities.

### Census of the Dog Population and Dog Vaccination

Dog population data on Bohol was first estimated from the 2006 census published by the Bureau of Agriculture Statistics (BAS). To secure data on the number and location of dogs throughout Bohol, the BRB initially conducted a house-to-house inquiry using a master list of households. The collected data reflected dog owner's name, number of dogs owned, whether they were confined, leashed or free-roaming, sex of each dog and total number of households. Dog population data was updated annually. To further regulate the possession of dogs, establish dog ownership, and facilitate the traceability of dogs involved in bite cases, the mandatory registration of dogs, with a corresponding fee collection, was implemented at the barangay level in accordance with the Provincial Ordinance. Dogs from households that were not able to afford the fees were also registered and the dog owners were given a promissory note and allowed a staggered payment. The collected registration fees were shared in a manner specified in the Provincial Ordinance as follows: 50% was retained in the barangay and 50% divided between the municipal and provincial treasury to support the sustainability of the entire rabies control program.

A parenteral mass dog vaccination program was initiated in August of 2007 with the Governor of Bohol proclaiming August 2007 as the synchronized rabies vaccination month. Vaccination teams were organized at the provincial and municipal levels. Provincial teams were assigned to oversee the vaccination activities and to ensure the presence and usage of cold chain equipment in every municipality. The Municipal Agricultural Officers (MAO) led the municipal vaccination teams composed of livestock technicians, Barangay Livestock Aides and other personnel duly designated by the MRPEC trained as dog vaccinators. Vaccination activities were supervised by the provincial and district veterinarians. The majority of the members of the vaccination team, (including those who administered the vaccine, or assisted in handling and tagging animals), received pre-exposure rabies vaccination. Team members who handled registration and collection of fees, or prepared reports did not come in contact with animals, and were not given pre-exposure rabies vaccination. All team members were briefed on proper rabies vaccination activities, provided vaccination supplies and paraphernalia, registration/health certificates and dog tags, recording forms and education campaign materials.

Mop-up dog vaccination campaigns targeting low coverage areas were conducted within six months following the initial mass vaccination campaign. Dogs not vaccinated during the scheduled synchronized mass rabies vaccination campaigns were accommodated upon special arrangement with the municipal vaccination teams.

A uniform dog tag, indicating vaccination, was securely fastened on a dog collar with the help of the assistant dog vaccinator. The tag was valid for one year from the date of vaccination and was replaced annually upon renewal of registration at which time a booster rabies vaccination was administered. Standardized dog vaccination report forms were consolidated and submitted to the BRB, the MAO and the BRPEP. The community was also encouraged to bring their cats for vaccination during the mass vaccination campaign.

### Dog Population Management and Movement Control

Dog population and movement control was implemented as part of the BRPEP and in compliance with the PNRA and the Animal Welfare Act. The dog population was managed by selective elimination of captured stray dogs, impounded dogs unclaimed within 3 days, and unmanageable dogs voluntarily submitted by owners [7].

Municipal rabies ordinances in the Philippines include a section on dog population management and designated a task force to perform this function. At the barangay level, socially acceptable procedures were discussed and widely disseminated throughout the community. Euthanasia procedures were initially conducted in accordance with Administrative Order No. 21 of the Department of Agriculture on the Code of Conduct in the Euthanasia for Pets/Companion Animals. During the third year of the project (2010), the purchase of a mobile veterinary clinic was funded by one of the partners and provided the opportunity to improve neutering, spaying, and euthanasia procedures. Additionally, partnerships were established with animal welfare organizations to improve dog population management practices to comply with recommended international standards.

### Clinical Management of Human Rabies Exposures

In 2007, prior to the launch of the BRPEP, two government-operated Animal Bite Treatment Centers (ABTC) were located on Bohol. As part of the BRPEP, four additional ABTCs, all privately operated, were established on the island to increase accessibility of timely post-exposure prophylaxis (PEP). Medical personnel were trained to administer rabies vaccines intradermally (ID) in order to provide effective PEP as economically as possible. Rabies biologicals were acquired either through the support of the DOH National Rabies Program, the investment of the private ABTCs, or direct procurement by the provincial government. Additionally, the government subsidized the purchase of PEP for indigents without sufficient financial resources, and for persons bitten by the dogs of responsible pet owners (those that had kept their animals registered and vaccinated) and responsible pet owners exposed to suspect rabid dogs. All exposed patients received PEP according to the WHO standard ID regimen and all adverse events were reported and managed accordingly [3]. Training on bite management and PEP for all district hospital doctors & nurses of the primary health care units was conducted in collaboration with the Research Institute for Tropical Medicine (RITM), the national rabies referral center of the Department of Health.

### Laboratory Diagnosis of Animals

When possible, animals involved in biting incidents were observed for 14 days. If the animal demonstrated clinical signs of rabies during the observation period, including behavioral change or illness, it was euthanized and submitted for testing at the Regional Animal Disease Diagnostic Laboratory (RADDL) located in the city of Cebu on the adjacent island province. Submitted specimens were analyzed using the direct fluorescent antibody test. Laboratory results usually available within 1–2 days were relayed to the OPV and the MRPEC who releases this to the victim or the immediate relatives. The sample testing was free of charge but the cost of transporting specimens was assumed by the BRPEP.

### Surveillance

Surveillance systems for both human and canine rabies cases were established in order to ensure immediate and reliable transfer of information and follow-up in the case of human or animal exposure to a confirmed rabid animal. The protocol for investigation and follow-up of all bite cases was established, and continues presently as follows: The reporting of bite cases is initiated at the BRB level when the bite victim presents at the Rural Health Center for consultation, first aid treatment and assessment; if referred, the patient attends the closest ABTC for PEP. In cases where multiple biting incidents have occurred or when the involved animal is suspected to be rabid, the MAO and the OPV are informed in order to monitor the status of the biting animal(s). In highly suspicious cases involving the death of a bite victim, the attending physician immediately reports the case to the Provincial Rabies Coordinator who in turn contacts the Quick Response Team (QRT) and the Provincial Surveillance Unit to initiate a field investigation. Contact tracing is conducted in coordination with the MRPEC, all human and animal contacts of the biting animal are assessed and, if deemed necessary, immediately given PEP (human patients) or a booster vaccination (animals). Movement of all animals involved in biting incidents is strictly monitored, the dog vaccination record at the barangay level is reviewed and mop-up vaccination is conducted.

### Monitoring and Evaluation

For planning purposes, estimating dog vaccination coverage, and evaluating the outcome of the project, a household survey was conducted to collect data on the owned dog population, knowledge, attitudes and practices (KAP), and data regarding the dog-human relationship. The survey was designed using questionnaires and cluster sampling procedures and collected data among 300 households [9], [10].

### Program Sustainability

Sustainability was ensured through the BRPEP activities on advocacy, general public awareness, child education, legislation, dog registration with fees, 100% of which was re-invested back into the BRPEP to establish a self-sustaining funding stream for the program. Sustainability was strategically built into the program through an integrated program interface with LGU officials, community leaders and other stakeholders to ensure community participation; establishment of a rabies diagnostic laboratory on the island for increasing further disease surveillance; creation of volunteer quarantine aides in coastal barangays to continually monitor the entry of new dogs from other provinces; and provision of annual award system to motivate excellence in program execution.

## Results

### Reduction in Incidence of Human and Animal Rabies Deaths

**Figure 2:**
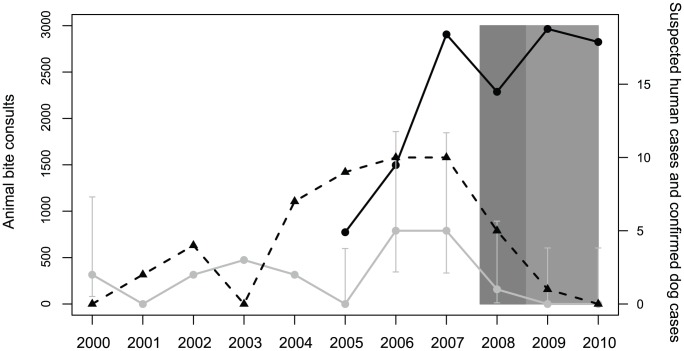
Number of suspected human cases (black triangles and black dashed line), reported animal bites (black dots and black solid line) and laboratory confirmed dog cases with 95% CI (gray dots and gray solid line), Bohol Province, 2000–2010. The shaded areas indicate the timing of round 1 (darker gray) and round 2 (lighter gray) mass dog vaccination.

Approximately 25% of all reported human deaths reported prior to the launch of the BRPEP occurred in children less than 15 years of age. After the initiation of the campaign, the number of human deaths dropped dramatically (Figure 2). The number of animal deaths also dropped with the last case of dog rabies being reported in April of 2011 (Figure 2).

### Legal Framework for the Program

The BPREP was strongly supported by the National Rabies Law and the local ordinances at the provincial and municipal level. There were 4,379 BRB members and 96 members of the MRPECs that received instruction through the paralegal training courses associated with the BRPEP. Local legal procedures and the utilization of paralegal forms reduced violations of the law and improved community compliance, resulting in fewer free roaming dogs to be eliminated.

### Communications Management

Guidelines at the provincial, municipal and barangay council levels ensured uniformity in the understanding of the program and resulted in synchronized efforts across the 47 municipalities, one city and 1,109 barangays of the province. The successful engagement of the local communities resulted in a community-focused program that increased the total workforce of the rabies elimination program from the initial 124 government paid staff prior to 2007, to about 15,021 in 2009. This included the participation of community volunteers and other local officials to augment the government employees. The KAP survey suggested that effective communications had promoted a sense of program ownership since 89% of the households surveyed believed that the BRPEP was good for their community and 73% of households no longer allowed their dogs to roam free.

### Information and Educational Campaign

As a result of the extensive IEC, there was a dramatic increase in the number of residents that acknowledged ownership of and registered their dogs. The community focused program also directly increased the number of bite victims that sought medical treatment after exposure. Increased dog bite reporting is a function of awareness, and the KAP survey revealed a high level of rabies knowledge among the general public: 94% claimed they had heard about rabies disease, 82% had knowledge about the local rabies program and 85% were aware of the rabies ordinances.

The school-based education program provided rabies lessons and modules that were integrated into the curriculum of all elementary schools throughout the province by year two of the program. This ensured early and continuous education for children considered most at risk. Rabies prevention education was expanded to all elementary schools, reaching over 182,000 children or 16% of the total provincial population. Additionally, 128 Rabies Scouters from three municipalities were mobilized as peer advocates for rabies prevention. Province-wide simultaneous World Rabies Day activities were observed in all elementary schools through the issuance of a directive from the Governor to the Education Department-Bohol and were highlighted by candle lighting ceremonies and offering of prayers for children that had died of rabies.

### Dog Population Control and Mass Vaccination

The 2006 BAS data, reported prior to the initiation of the BRPEP, indicated that the dog population was 168,161 of which 2.8% were estimated to be vaccinated. The total dog population on Bohol in 2007, as more accurately reported by the house to house survey, was 100,752 dogs, a significantly lower number than the 2006 data. For 2007 and 2008, dogs registered and paid for by owners totaled 44,516 and 53,692 respectively.

Vaccination coverage of the dog population increased from 2.6% in 2007 to 44% in 2008. In 2009, after a second BRB survey, the dog population was recorded to be 76,407, of which 70% were registered and vaccinated. This coverage was obtained despite the introduction of mandatory registration fees. Reasons why the remaining 30% of dogs were not vaccinated included: owner absent during the vaccination campaign (20%); owner unable to restrain dogs (18%); dogs were thought to be too young (18%); and unable to afford fees (5%).

In 2008, the LGU task forces eliminated 2,677 free-roaming dogs and 705 in 2009. The same household survey revealed that unowned dogs were always present in the neighborhood, the median was 3.0 with a range of 1–18 [10]. Backed by continuous public advocacy, LGUs began adopting the paralegal measures where the focus was to penalize dog owners violating the Rabies law. The KAP survey revealed that 34.5% of the male dogs, accounting for 66.3% of the total dog population, were castrated; and 73% of the households no longer allowed their dogs to roam freely day and night.

### Clinical Management of Rabies Exposures

Through the BRPEP, a specific protocol was established to ensure that all persons exposed to potentially rabid animals received appropriate and prompt PEP. As part of the program, the number of animal bite treatment centers/clinics (ABTCs) was increased from two to six. Between 2007 and 2009, 8,158 patients received PEP through the provincial and private ABTCs (Fig. 2). Of these patients, 52.5% had WHO category III wounds. The number of patients that received PEP increased annually between 2007 and 2009. Animal bite reporting forms indicated that the number of potentially exposed children less than 15 years of age comprised 47.27% of all patients reporting to an ABTC between 2007–2009. Of the recorded animal bites during this time period, 95.78% (7,812) of all potential exposures were reported to have been caused by dog bites.

### Diagnosis of Rabies

A total of 148 dog head samples were tested by FAT between 2007 and 2010. Of the samples submitted, 4% were confirmed positive for rabies. All of the 134 samples submitted for testing in 2009 and 2010 were confirmed negative for rabies. In April 2011, one dog sample was confirmed positive in Ubay Municipality, the same area as where a suspected human case had been reported in November 2010. The dog was <1 yr old and unvaccinated and had bitten two people, including a 7 year old. All persons exposed to the dog received PEP and a mop-up vaccination campaign and a house-to-house educational program were both initiated within the week.

One suspect human rabies case was reported in November 2010. The young girl had been bitten on the upper left thigh on 18 October 2010 by a nursing dog after she had provoked it with a stick. The wound had not been cleaned and the patient was taken to a traditional healer the same afternoon as the bite occurred. On 23 November, the patient experienced intermittent low-grade fever and was given paracetamol, on 25 November, the patient manifested hydrophobia. On 26 November the patient was brought to a hospital in Ubay and was prescribed paracetamol and sent home. The patient died at home the next day and was buried before samples could be secured for testing. Following the provincial program SOP, a joint investigation by the provincial health, veterinary and local government staff was conducted within 72 hours of the time of death. Contact tracing and interview of key informants including the family and neighbors was immediately conducted by the investigating team but revealed no possible related human and animal cases in the locality and neighboring villages. There was immediate initiation of a mop up dog vaccination, intensified public awareness campaign and dog catching and testing for rabies.

In 2010, the Provincial Government allocated funds for the establishment of a Provincial Rabies Diagnostic Laboratory. In preparation for its actual operation, a training course for rabies diagnoses using the direct rapid Immunohistochemistry test (dRIT) was conducted for the veterinary staff by the US Centers for Disease Control and Prevention (CDC) and RITM. A total of 12 local government veterinarians were trained. The CDC subsequently signed a Material Transfer Agreement with the provincial government for the equipment, supplies and specimen sharing. Thereafter, testing tissue samples using the dRIT began with validation by RITM.

### Surveillance and Disease Monitoring

Reporting of suspected dog rabies was not routinely conducted prior to the initiation and launch of the BRPEP. Between 2002 and 2006, sample submission and confirmation of rabies infection was minimal (Table 1). During the program implementation years, surveillance for rabies in animals was enhanced through the introduced system of observation of biting animals that were not immediately tested for rabies.

**Table 1:** Dog population, vaccination, rabies diagnosis, elimination and movement data, and human rabies and animal bite cases, Bohol, Philippines, 2000–2011

	2000	2001	2002	2003	2004	2005	2006	2007		2008		2009		2010	2011
Mass dog vaccination campaigns								*	**Aug 07–Jul 08**		**Aug 08–Sept 09**		**Oct 09–Dec 10**		**Jan–Jun 11**
1 Dog population estimate	128,194	*	*	*	*	151,301	168,161		100,752		76,407		73,547		74,962
2 Vaccinated dogs	100,157	24,930	20,350	38,700	19,270	3,824	4,801		44,516		53,739		35,885		19,852
Dog vaccination coverage	86%	*	*	*	*	3%	3%		44%		70%		49%		26%
Brain tissue samples submitted	0	0	9	13	12	3	14	11		4		113		21	60
for diagnosis															
Confirmed dog cases	2	*	2	3	2	0	5	5		1		0		0	1
3 Annual incidence of confirmed dog cases per 100000 (95% CI)	0.16 (0.04–0.57)	*	*	*	*	0 (0–0.25)	0.30 (0.13–0.70)	0.50 (0.21–1.16)		0.13 (0.01–0.74)		0 (0–0.52)		0 (0–0.52)	0.13 (0.01–076)
Suspect human cases	0	2	4	0	7	9	10	10		5		1		0	0
Annual incidence of suspect human cases per 100000	0	0.17	0.33	0	0.57	0.72	0.70	0.77		0.37		0.07		0	0
Animal bite bite consults	*	*	*	*	*	773	1497	2906		2287		2965		2824	1560
Dog elimination by LGUs						*	*	*		2677		705		281	408
Dogs brought into Bohol								1806		2238		2363		2795	684
Dogs taken out of Bohol								3365		3498		3643		4017	990

1 Source for dog population size estimation: Municipal Agriculture Office; Bureau of Agricultural Statisctics; House to house survey by barangay livestock aid (BALA).

2 Method of dog vaccination campaign: House to house only; *Mixture of central point and house to house.

3 After 2007, incidence was calculated using the more conservative dog population estimate e.g. for 2008, 100,752 dogs was used.

* N.B. Omitted data means data is missing because it was not collected, and therefore is not necessarily zero.

### Program Sustainability

Two rounds of compulsory registration and the collection of yearly registration fee of a total of 95,167 dogs between 2007–2009 established dog ownership and traceability of biting incidents, and enabled the program to generate revolving funds in the amount of 105,740 US$.

Annual data from the inter-provincial records of dog movement indicated that the number of dog entries recorded increased each year (Table 1). Recorded data for the same time span indicated that the number of dogs leaving Bohol each year also increased (Table 1). Dogs newly introduced into the community from new births or importation were targeted during routine vaccination campaigns.

## Discussion

The three-year BRPEP, initially launched in 2007, successfully achieved the goal of establishing a sustainable program with the aim of eliminating human and canine rabies throughout the province of Bohol contrary to the program that was initiated in 2000. The lack of a sustainable program in 2000 inevitably allowed canine rabies to spread throughout Bohol and the number of human rabies cases began to steadily increase. As 25% of the rabies deaths were in children, there was a need to especially focus on increasing education in this age group. Support from several stakeholders, including the Governor of Bohol, resulted in an agreement on a new action plan to tackle the increasing rabies problem. From the early planning stages of the BRPEP, all stakeholders and partners committed to building an intersectoral rabies control program on Bohol that would be self-sustaining when outside funding channels were no longer available.

The BRPEP was strongly supported by existing national and local laws and ordinances including the PNRA. However, actual implementation of the program required an understanding of the need to implement the existing laws. This was achieved by increasing educational awareness across all levels of society, particularly focusing on how enforcement of the laws would improve the daily lives and the public health of the community. From the beginning of the program it was understood that only acceptance and ownership of the program at the community level would achieve sustainable and effective field operation. Thus, the massive social mobilization component of the BRPEP, including increasing awareness and improving citizen involvement in rabies prevention activities, were major factors in creating a successful community-based rabies control and prevention program on Bohol.

The support and involvement of the Department of Education was critical for the successful implementation of the early childhood education about rabies prevention. The full integration of lessons on rabies and responsible pet ownership into the curriculum of all elementary schools throughout the province was the first program of its kind in the country. It demonstrated that working with the education sector was an effective strategy to improve awareness in the young population who are at most risk to rabies exposure.

The surveillance of animal rabies prior to the initiation of the BRPEP was very poor. There were no diagnostic facilities on the island and accurate data regarding the number and species of animals infected with rabies throughout the province was unavailable. The importance of submitting suspected dogs for laboratory examinations was not appreciated and sending these samples for testing was costly resulting in few samples being submitted. The program improved reporting of suspected canine rabies cases through the active participation of the BRBs, rural health centers and the ABTCs. Prior to the program, dogs were commonly raised without confinement and stray dogs were a part of daily life. Dogs were responsible for 98% of the human rabies cases. Therefore, controlling the dog population and dog movement to achieve a feasible regular vaccination program and prevent the spread of rabies were both deemed necessary. Although stipulated in the Anti-Rabies Act of 2007, that “LGU should enforce dog impounding activities and field control to eliminate stray dogs”, strong political will and sufficient financial resources required to comply with the law were lacking [7]. In order to generate more community support and overcome cultural barriers regarding responsible pet ownership, the BRPEC increased community advocacy about rabies as a disease, its public health importance, legal implications and how everyone in the community could help to eliminate the disease throughout the province.

For planning and evaluation of control and vaccination activities and acceptable methods, accurate information on dog population density and dog-human relationships was extremely valuable. Data collected by the relatively simple field methods employed in this project sufficed for planning cost-effective dog rabies control campaigns throughout the province. Dog population and ecology data collected will also be useful in planned epidemiological analyses of canine rabies in Bohol.

The dog population on Bohol was decreased over four years by various methods including: strengthening of the national and provincial rabies laws that supported the community task forces; owners voluntarily reducing the number of dogs they retained; tighter controls on animal movement; increased clinics for spaying and neutering; operation of dog pounds/cages; and strengthening of veterinary quarantine services that ensured only vaccinated dogs with registration cards entered and left the province. Province-wide paralegal trainings also reinforced responsible dog ownership and reduced the stray dog population. The component on dog population management and movement control was effectively implemented only during the second year as pressure mounted from the community itself, in response to the BRPEP.

In any large rabies control program high operational costs become significant challenges not only for the provincial government but also for each partner community. To help defray the cost of the program and improve community involvement, the BRPEP enlisted volunteers throughout the province. Volunteers are well-known and respected by their neighbors, have a thorough knowledge of the local settings, particularly in remote areas, and are motivated to serve their own communities. Through volunteerism, the number of people involved in implementing the rabies control program was increased from 124 to over 15,000.

The Bohol Rabies program implementation between 2007 and 2010, required a budget allocation of approximately USD 450,000 and therefore resource mobilization was essential. Funding was supplied by several different program stakeholders including local and national governments and partner NGOs. At the community level, funding was generated through the collection of dog registration fees. This was in compliance with the National Rabies Law and the Provincial Local Ordinance. Although the main purpose of the registration fee was to regulate the keeping of dogs at the household level, fees collected have also served as a resource for operational funds since registered dogs are entitled to a free rabies vaccination, a dog tag with collar and a registration card. The average program cost per vaccinated dog was USD 1.62. The total community funds generated since the initiation of the program was enough for the next annual program budget.

This article reports the overall results from the model rabies eradication program in an island province in the Philippines. Human deaths and canine cases have been dramatically decreased and surveillance improved. The success of the program was achieved through empowerment of the local communities and the use of the one health intersectoral approach to rabies control. The program was achieved through the joint efforts and shared resources of local and national government, various sectors of public health, animal health and agriculture, environment, legislation and policy, as well as non-government partners.
